# Successful Management of a Gustilo-Anderson Type I Open Comminuted Tibial Fracture Using Ilizarov Circular External Fixation: A Case Report

**DOI:** 10.7759/cureus.106921

**Published:** 2026-04-12

**Authors:** Md Sulaiman

**Affiliations:** 1 Orthopaedic Surgery, Trauma General Hospital and Diagnostic Center, Brahmanbaria, BGD

**Keywords:** circular external fixation, fracture healing, gustilo-anderson fracture, ilizarov fixator, open tibial fracture

## Abstract

Open tibial fractures are challenging injuries due to limited soft tissue coverage, contamination, and the risk of infection or delayed union. The Ilizarov circular external fixation system provides stable fixation while preserving periosteal vascularity, minimizing further soft-tissue disruption, and allowing early weight bearing. We report a case of a 50-year-old man who sustained a Gustilo-Anderson type I open comminuted tibial fracture following a road traffic accident. Initial management included wound irrigation, sterile dressing, immobilization, and intravenous antibiotics. Definitive treatment was performed with irrigation, debridement, and application of a four-ring Ilizarov circular external fixator under spinal anesthesia. Partial weight bearing was initiated as tolerated and gradually progressed to full weight bearing during follow-up. Follow-up radiographs demonstrated satisfactory callus formation and subsequent radiological union with maintained alignment. The patient achieved independent ambulation with satisfactory knee and ankle motion, and a minor pin-site infection consistent with Checketts-Otterburn grade 1 was observed during follow-up. This case highlights a practical decision-making point: in selected Gustilo-Anderson type I open comminuted tibial fractures, primary Ilizarov fixation may be preferred when preservation of fracture biology, avoidance of further soft-tissue disruption, and early weight bearing are major treatment priorities.

## Introduction

Open tibial fractures represent a significant challenge in orthopedic trauma because of the high risk of infection, delayed union, and soft tissue complications. Circular external fixation, particularly the Ilizarov method, has been widely used for complex tibial fractures, especially when preservation of soft-tissue biology and stable fixation are key treatment priorities [1].

The Ilizarov technique is based on the principle of tension-stress, which induces osteogenesis through controlled mechanical loading and supports bone regeneration and soft-tissue healing [2]. Several studies have reported successful outcomes using Ilizarov circular external fixation in the treatment of complex tibial fractures, particularly when soft tissue conditions limit the use of internal fixation [3].

Proper classification of open fractures is essential for guiding treatment decisions, as it directly influences antibiotic selection, surgical timing, and fixation strategy. The Gustilo-Anderson classification remains the most widely used system for grading open injuries and estimating prognosis [4].

In this report, we present a case of a Gustilo-Anderson type I open comminuted tibial fracture successfully managed with Ilizarov circular external fixation with satisfactory clinical and radiological outcomes.

## Case presentation

**Figure 1:**
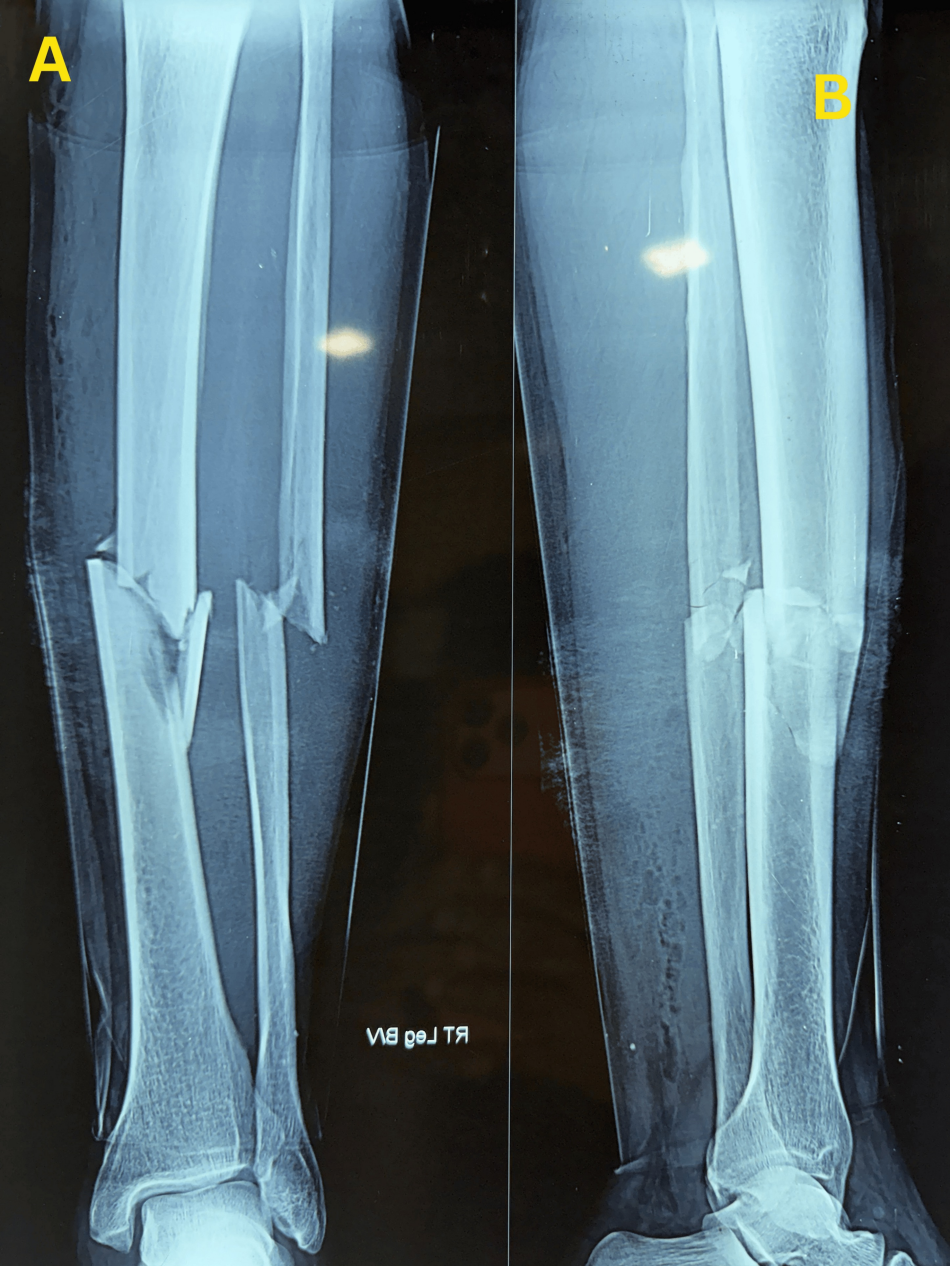
Preoperative radiographs of the right tibial fracture (A) Anteroposterior radiograph showing a comminuted midshaft fracture of the right tibia. (B) Lateral radiograph demonstrating displacement of the fracture fragments.

A 50-year-old man presented to the emergency department following a road traffic accident with pain, swelling, and an open wound over the right leg. Clinical examination revealed tenderness, deformity, and a clean open wound measuring approximately 2 cm over the anterior aspect of the right leg, consistent with a Gustilo-Anderson type I open tibial fracture. Distal neurovascular status was intact. Radiographs demonstrated a displaced comminuted mid-diaphyseal fracture of the right tibia with an associated mid-diaphyseal fibular fracture. The injury was classified as a Gustilo-Anderson type I open tibial fracture (Figure 1).

**Figure 2:**
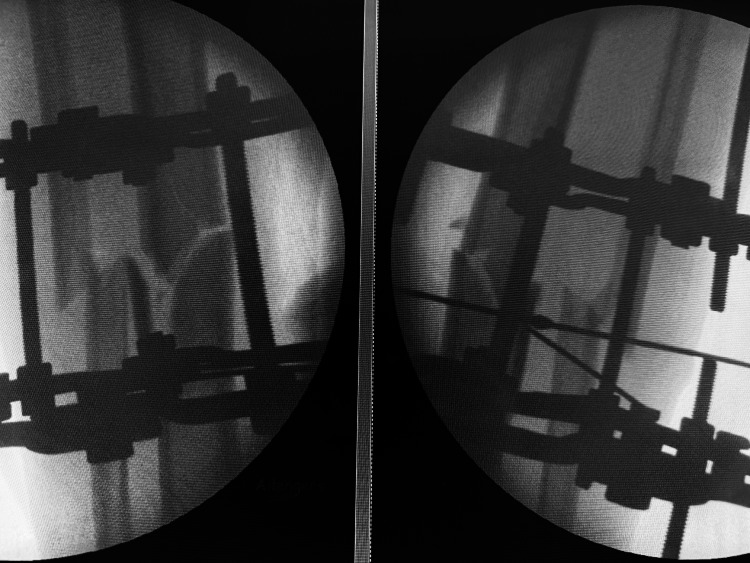
Intraoperative C-arm fluoroscopic image during application of Ilizarov circular external fixator Intraoperative C-arm fluoroscopic image demonstrating application of a four-ring Ilizarov circular external fixator for stabilization of the midshaft tibial fracture. Tensioned wires and connecting rods were used to achieve stable fixation and maintain fracture alignment.

Initial management included wound irrigation, sterile dressing, immobilization with a temporary splint, tetanus prophylaxis, and intravenous antibiotics. After appropriate preoperative preparation, the patient was taken to the operating room for definitive management. Under spinal anesthesia, thorough irrigation and debridement were performed, followed by application of a four-ring Ilizarov circular external fixator using tensioned 1.8 mm transosseous wires and connecting rods to achieve stable fracture fixation. An olive wire was additionally used to enhance compression and rotational stability (Figure 2).

**Figure 3:**
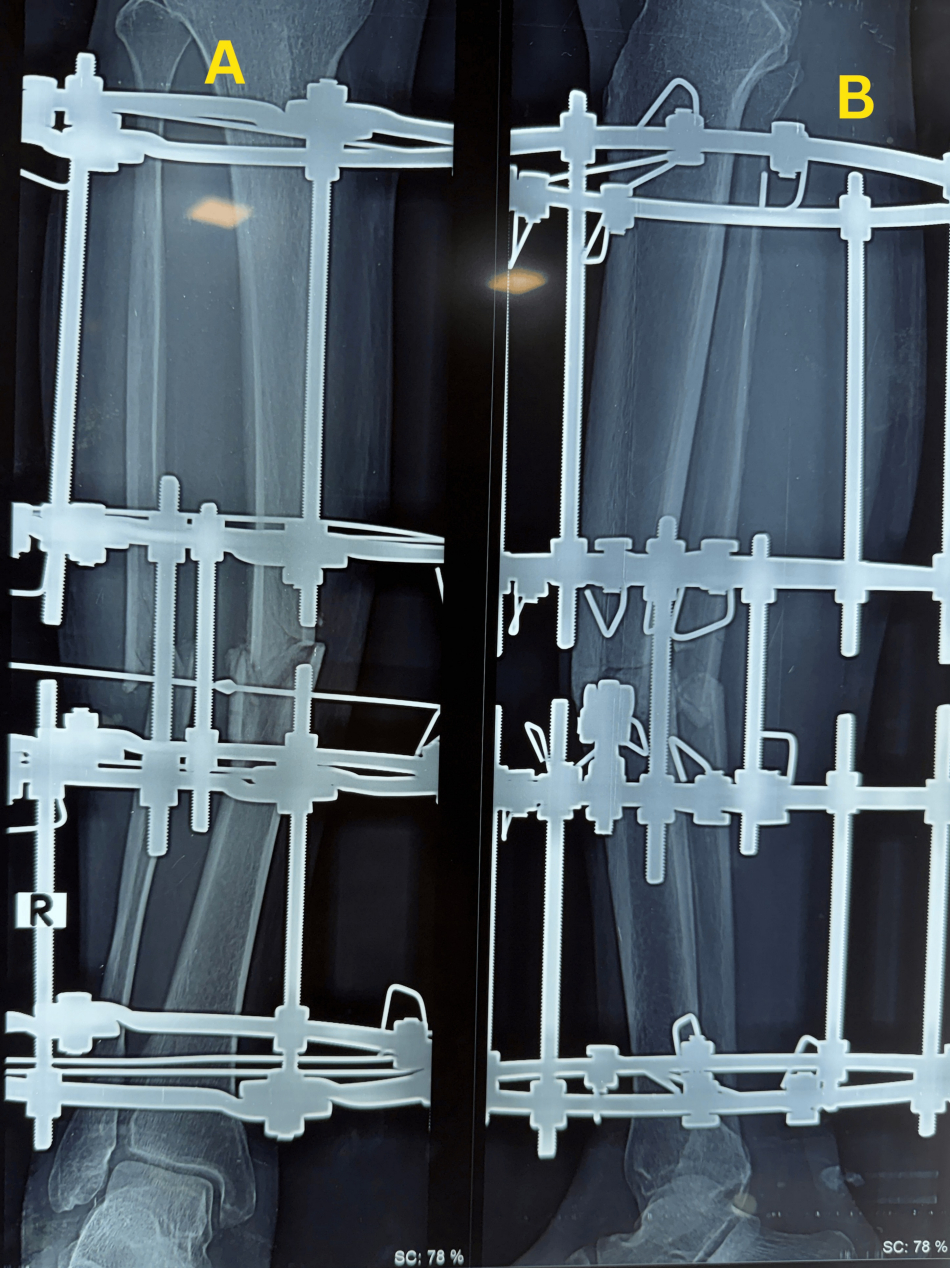
Follow-up radiographs demonstrating callus formation at the tibial fracture site (A) Anteroposterior view demonstrating progressive callus formation at the midshaft of the right tibia. (B) Lateral view showing maintained fracture alignment with developing callus across the fracture site.

**Figure 4:**
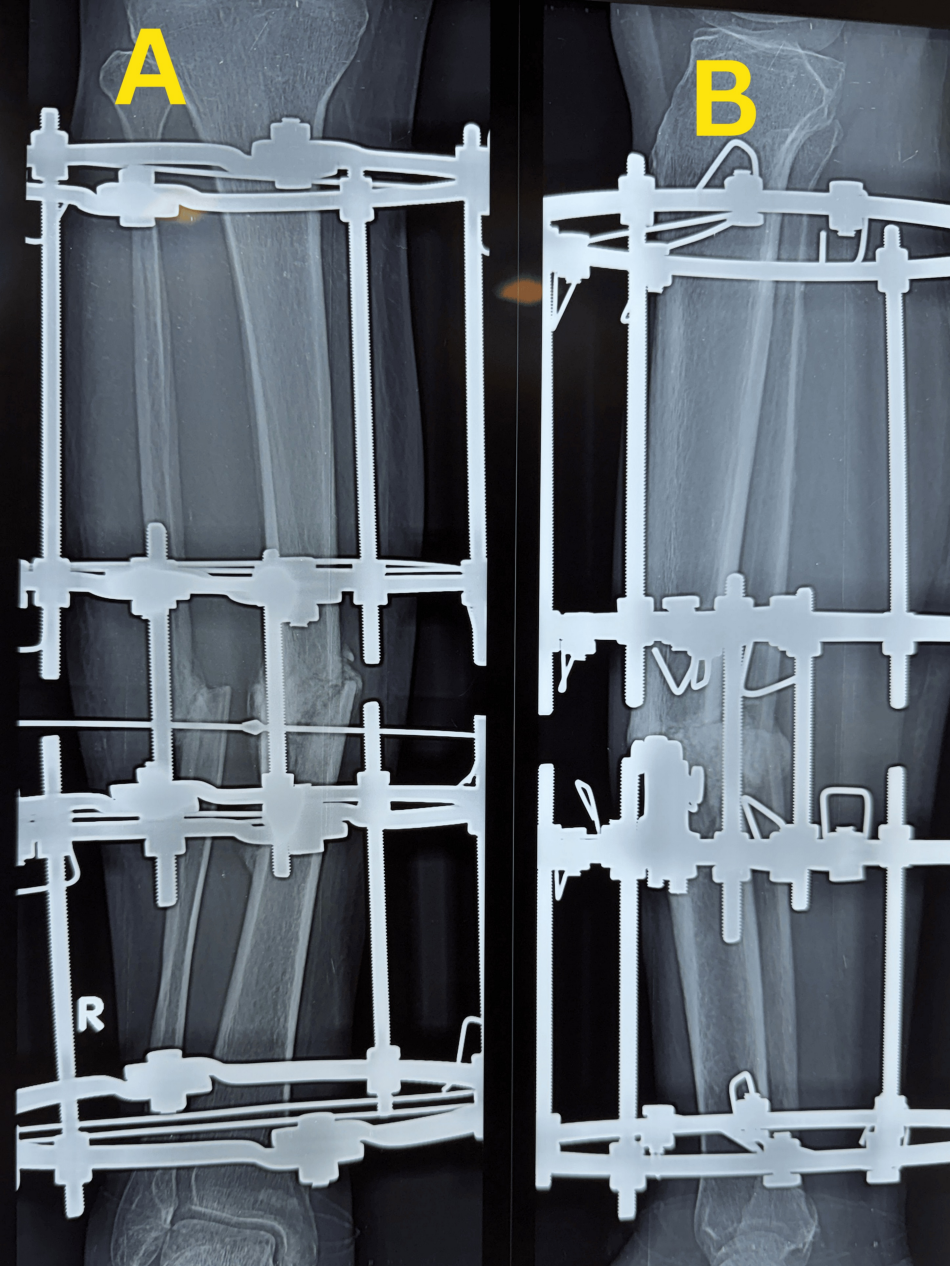
Follow-up radiographs demonstrating fracture union with maintained alignment Radiological union of the midshaft right tibial fracture is demonstrated by bridging callus across at least three cortices, with maintained alignment. (A) Anteroposterior view. (B) Lateral view.

**Figure 5:**
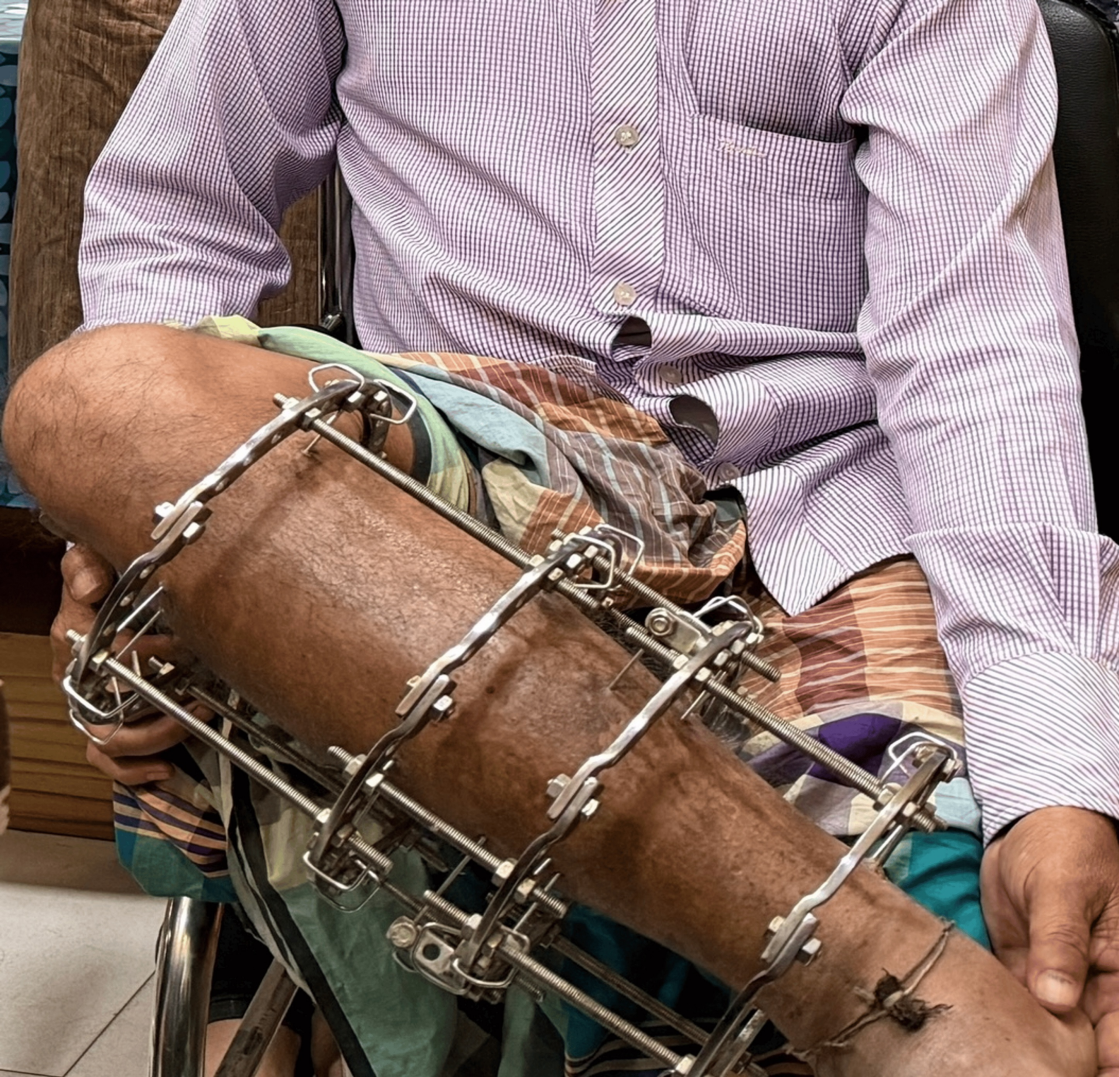
Clinical photograph showing Ilizarov circular external fixation applied to the right tibia Clinical photograph demonstrating the Ilizarov circular external fixation construct applied to stabilize the midshaft fracture of the right tibia during follow-up.

Postoperatively, partial weight bearing was initiated as tolerated and gradually progressed during follow-up. Serial radiographs demonstrated progressive periosteal and endosteal callus formation with bridging across at least three cortices while maintaining fracture alignment (Figure 3). Follow-up radiographs taken on 7 March 2026 demonstrated fracture union with maintained alignment (Figure 4). At follow-up, the patient showed satisfactory clinical and radiological improvement with good functional recovery (Figure 5).

## Discussion

Open tibial fractures are associated with significant morbidity due to compromised soft tissue coverage and the risk of infection. Early debridement, appropriate antibiotic therapy, and stable fracture fixation are key principles in the management of these injuries. The Gustilo-Anderson classification remains a valuable tool for assessing injury severity and guiding treatment strategies [4].

Circular external fixation using the Ilizarov method offers several advantages in the management of tibial fractures. It provides stable fixation while preserving the blood supply to fracture fragments and surrounding soft tissues. Previous studies have shown that circular external fixation, including the Ilizarov method, can achieve high union rates in tibial fractures, although pin-tract sepsis remains a recognized complication [1].

The Ilizarov technique functions based on the principle of tension-stress, which stimulates osteogenesis and promotes both bone and soft tissue regeneration. The modular structure of the Ilizarov frame also allows gradual correction of alignment and controlled compression at the fracture site, contributing to improved stability and fracture healing [2].

Recent reports have demonstrated successful outcomes using Ilizarov fixation in complex open tibial fractures requiring reconstruction and stabilization [3]. In the present case, application of a circular external fixator with tensioned wires and an olive wire provided stable fixation, maintained alignment, and allowed early weight bearing, ultimately resulting in satisfactory clinical and radiological recovery.

Although intramedullary nailing is often preferred for open tibial fractures because of lower reported rates of superficial infection and malunion, circular external fixation remains a useful alternative in selected cases, with similar reported rates of deep infection, delayed union, and nonunion [5]. In the present case, Ilizarov circular external fixation provided stable fixation while preserving fracture biology and minimizing additional soft-tissue disruption, allowing satisfactory fracture union and functional recovery.

## Conclusions

Primary Ilizarov circular external fixation can be an effective option in selected Gustilo-Anderson type I open comminuted tibial fractures when preservation of fracture biology, avoidance of further soft-tissue disruption, and early weight bearing are key treatment priorities. In this case, it achieved stable fixation, satisfactory radiological union, and good functional recovery. This case highlights the practical value of Ilizarov fixation as a biologically respectful treatment strategy in carefully selected open comminuted tibial fractures.

## References

[1] Marais LC, Ferreira N (2018). Circular external fixation in the management of tibial plateau fractures in patients over the age of 55 years. SA Orthop J.

[2] Kouzelis A, Vrachnis IN, Vris A, Zampakis P, Kokkalis ZT, Panagopoulos A (2020). A novel treatment of a 65-year-old woman with a neglected type IIIB open fracture of the tibia with inadequate soft tissue coverage and periosteal stripping requiring an Ilizarov approach to bone and soft tissue lengthening and reconstruction: a case. Am J Case Rep.

[3] Alpharian GT, Robiady YS (2024). Treatment of grade 3B open tibia fracture by segmental resection and bone transport: A case report and literature review. Int J Surg Case Rep.

[4] Yim GH, Hardwicke JT (2018). The evolution and interpretation of the Gustilo and Anderson classification. J Bone Joint Surg Am.

[5] Jeremić D, Rajovic N, Gluscevic B (2023). Updated meta-analysis of randomized controlled trials comparing external fixation to intramedullary nailing in the treatment of open tibial fractures. Medicina (Kaunas).

